# Machine learning with PROs in breast cancer surgery; caution: Collecting PROs at baseline is crucial

**DOI:** 10.1111/tbj.13804

**Published:** 2020-03-11

**Authors:** Laurentine S. E. van Egdom, Andrea Pusic, Cornelis Verhoef, Jan A. Hazelzet, Linetta B. Koppert

**Affiliations:** ^1^ Department of Surgical Oncology Erasmus MC Cancer Institute Rotterdam The Netherlands; ^2^ Department of Plastic and Reconstructive Surgery Patient‐Reported Outcomes, Value & Experience (PROVE) Center Brigham and Women’s Hospital Boston MA USA; ^3^ Department of Public Health Erasmus MC University Medical Center Rotterdam The Netherlands

**Keywords:** breast cancer surgery, machine learning, patient‐reported outcomes

## Abstract

As high breast cancer survival rates are achieved nowadays, irrespective of type of surgery performed, prediction of long‐term physical, sexual, and psychosocial outcomes is very important in treatment decision‐making. Patient‐reported outcomes (PROs) can help facilitate this shared decision‐making. Given the significance of more personalized medicine and the growing trend on the application of machine learning techniques, we are striving to develop an algorithm using machine learning techniques to predict PROs in breast cancer patients treated with breast surgery. This short communication describes the bottlenecks in our attempt to predict PROs.

1

Improvement in early detection and treatment of breast cancer has resulted in increased long‐term breast cancer survivors.^1^ The cornerstone of breast cancer management still is surgery. In breast cancer surgery, equal survival rates are achieved, irrespective of type of surgery performed.^2^, ^3^, ^4^ However, breast cancer surgery can adversely affect women's psychological health and health‐related quality of life (HRQoL). Prediction of long‐term physical, sexual, and psychosocial outcomes is therefore very important in treatment decision‐making.

Patient‐reported outcomes (PROs) come directly from the patient without interpretation by a health care provider and reflect aspects of health, quality of life, and related constructs.^5^ The routine collection of PROs has been implemented in many health institutions,^6^, ^7^, ^8^, ^9^, ^10^ and it is clear that PROs have an important role in today's clinical practice. Collaboration of the International Consortium for Health Outcomes Measurement (ICHOM) with several other health care institutions worldwide has resulted in the development of a Standard Set for breast cancer outcomes.^10^ Within this outcome set, patient‐reported outcome measures (PROMs) are pivotal and accounting for 75% of the outcomes evaluated.^10^

PROs can help facilitate in shared decision‐making through informing treatment decisions and setting expectations. The ability for patients to understand what other patients with breast cancer experienced after surgery is thereby vital.

Predictive modeling is not new to medicine. In clinical medicine, a multivariable prediction model combines information from multiple predictors to predict the probability of or risk for a specific disease or outcome.^11^ Predictive modeling has the purpose of informing patients and guiding clinicians in decision‐making on treatment decisions. The majority contains prediction of patient outcomes focused on cancer survival and risk of cancer recurrence/local control,^12^, ^13^, ^14^ but little has been done to predict PROs into the future. Moreover, to our knowledge, there are no tools available focusing on predicting HRQoL outcomes after breast surgery into the future. Given the significance of more personalized medicine and the growing trend on the application of machine learning techniques, our breast cancer team is striving to develop an algorithm using machine learning techniques to predict PROs in breast cancer patients treated with breast surgery.

We aimed to develop and validate a simple prediction model for improvement of HRQoL after breast cancer surgery using data from three PRO questionnaires as proposed in the ICHOM Standard Set for Breast Cancer, namely the EORTC QLQ‐C30 and EORTC QLQ‐B23, and the BREAST‐Q (postoperative modules). To this end, a retrospective cohort collected and described previously^6^ was used. This cohort contained 764 female patients with breast cancer (pTis‐3N0‐3M0) who underwent breast cancer surgery between January 2005 and September 2016 at Erasmus MC Academic Breast Cancer Center, Rotterdam, the Netherlands. Data on patient characteristics, age, date and type of surgery, tumor morphology, TNM staging (7th edition^15^), hormonal status, HER2 status, *BRCA* 1/2 gene mutation status, local recurrence, second primary breast cancer, details regarding chemotherapy and/or immunotherapy and endocrine therapy, radiotherapy, and follow‐up were available. Machine learning (ie, general linear model regression (GLM), support vector machines (SVM), single‐layer artificial neural networks (ANN), and deep earning (DL))^16^ was used to jointly study presurgical prognostic variables relating to age, medical status, tumor characteristics, and possible (neo)adjuvant treatment indications/treatment characteristics. Unfortunately, a lack of relationship was found between outcome variables and their predictors, meaning that the accuracy reflected just the population prevalence of the outcomes. Machine learning models have an immense number of parameters that must be either learned using data or set manually by the researcher.^17^ By combining variables in a reduced number of dimensions, we tried to help the analysis, but this did not yield substantial changes and required days of computational time.

During the process, some crucial obstacles were identified, which stagnated the development of a machine learning model in this dataset. This included the cross‐sectional design, the lack of baseline PROs, and the relative small sample size. Given the increase in the use of machine learning techniques in medical research and the, worldwide, desire to predict and influence PROs after breast surgery, we believe it is important to draw attention to our findings.

Machine learning describes the use of computer algorithms that learn nonlinear associations retrospectively from the data to estimate risk of a specific outcome. Even though machine learning is increasingly used in medical research,^18^, ^19^, ^20^ success is not always guaranteed. As with any method, a good understanding of the problem and an appreciation of the limitations of the dataset is important. Also crucial is an understanding of the assumptions and limitations of the algorithms being applied. If a machine learning experiment is properly designed, with correctly implementation and validated results, there usually is a good chance of success.

Although we used patient and treatment characteristics, and outcomes of interest to both patients and clinician (ie, validated PROMs as proposed in the ICHOM Standard Set for Breast Cancer), there were some important limitations in using the existing dataset.^6^ With 764 breast cancer patients, the study was relatively large, although for machine learning techniques probably not large enough. The size of the dataset is one of the most common limitations noted in studies reporting machine learning techniques.^14^ The dataset needs to be sufficiently large, which allows sufficient partitioning into training and testing sets, leading to reasonable validation of the estimators^14^ in order to enhance the generalizability of the predictive model.

The most important limitation however is the cross‐sectional design of the dataset, meaning the absence of baseline PROs. Traditional methods for evaluating PROMs look at the change over time, using the baseline compared with the end point. Enabling comparison with preoperative PROs is expected to reflect the influence of different treatments on HRQoL outcomes better than a single score obtained following treatment. One explanation probably is the fact that not every individual patient will score their breasts to the highest possible level at baseline. Although preoperative PROs were not available, all known other potential predictors were assessed, except for socioeconomic status (which cannot be easily obtained in the Netherlands for privacy reasons). The next step toward further validation of this approach to prediction would be to work with a more complete dataset, including baseline PROs and lifestyle measures. The research team has secured a prospective dataset over a longer time frame, but this dataset currently consists of a small number of patients. Since PROM collection is considered standard of care at our institute nowadays,^9^ in combination with a regional and international collaboration, this cohort will be progressively enlarged over time. There are plans in place to develop and test the performance of the machine learning techniques in this dataset in the near future. However, the above‐described study was a valuable first step toward modeling PROM data for use in breast cancer surgery. Once developed, the model could have potential for use outside breast surgery because similar sets are used in other diseases. But, as also suggested by Beam et al,^17^ the challenges and obstacles to reproducibility of machine learning techniques must be carefully considered to ensure the validation, safety, and effectivity of these new class of prediction tools.

In conclusion, using machine learning methods, we endeavored to develop a clinical prediction model for PROs after breast surgery. Clinicians could use information on the level of patient HRQoL outcome improvement, when counseling patients about the (prognostic) outcomes of breast cancer surgery, allowing patients to be more involved in their treatment decision. To actually realize an effective clinical prediction model, information regarding patients’ starting position is crucial. This emphasizes the urgent need of collecting PROMs at baseline, leading to the opportunity of predictive modeling on PROMs in breast cancer surgery in the future.
